# Epigenetics and Metabolism in Health and Disease

**DOI:** 10.3389/fgene.2018.00361

**Published:** 2018-09-18

**Authors:** Evangelia Tzika, Tobias Dreker, Axel Imhof

**Affiliations:** ^1^4SC AG, Translational Pharmacology, Munich, Germany; ^2^Faculty of Medicine, Ludwig Maximilians University of Munich, Munich, Germany; ^3^Protein Analysis Unit (ZfP), Biomedical Center, Ludwig Maximilians University of Munich, Munich, Germany

**Keywords:** epigenetics, metabolism, cancer, diabetes, HDACs, HATs, methyltransferases, demethylases

## Abstract

In the next 10 years, one billion people are estimated to suffer from disabling consequences of metabolic disorders, making them the number one noncommunicable disease on a global scale by 2030. Lots of risk factors such as dietary intake, lack of exercise and other life style behaviors are considered to play a role in the development of metabolic disorders. Despite the efforts that have been undertaken to unravel their potential causes, the underlying molecular mechanisms remain elusive. Evidence suggests that the pathogenesis involves changes on chromatin and chromatin-modifying enzymes, which can contribute to a persistent dysregulated metabolic phenotype. Indeed, a rising number of studies links epigenetic alterations with the diagnosis and prognosis of metabolic disorders. A prerequisite for exploiting these findings for pharmacological intervention is a detailed understanding of how differential epigenetic modifications control cell metabolism. In this mini review, we summarize the recent advances in uncovering the interplay between epigenetics and metabolic pathways on a cellular level and highlight potential new avenues for alternative treatment strategies.

## Introduction

**FIGURE 1:**
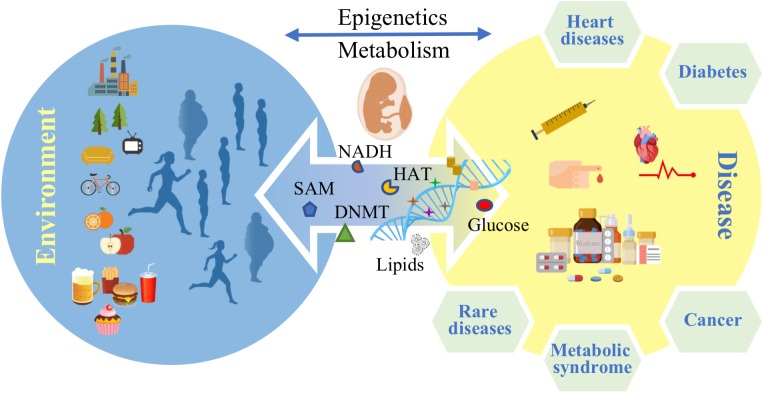
Environmental factors affect epigenetics and metabolism and control disease predisposition in later life stages.

Noncommunicable diseases (NCDs) are the leading cause of morbidity and mortality worldwide (WHO, 2017). Metabolic diseases constitute the greatest part of NCDs and according to the International Diabetes Federation (IDF) atlas for 2017, more than 693 million people will be suffering from diabetes by 2045 (IDF, 2017). Obesity and cardiovascular diseases are rising (WHO, 2014) with the prevalence of obesity projected to 1 billion people worldwide by 2030 (Kelly et al., 2008). A group of risk factors such as high fasting blood sugar, high blood pressure, low HDL cholesterol level and others are defined as the “metabolic syndrome," which is thought to predict or increase the risk for diabetes and heart diseases (NIH, 2016). These data emphasize the urgent need to find better prevention methods and therapies for the metabolic syndrome. However, many questions remain unanswered about the pathology of the metabolic diseases and factors such as life style behavior like nutrition habits and training routine are considered to play a role in the development of the diseases across different patients (Jumpertz von Schwartzenberg and Turnbaugh, 2015; Zeevi et al., 2015; Khera et al., 2016; Phillips, 2016). The molecular mechanisms that frame the influence of metabolism on possible gene deregulation patterns, underpinning causality and onset of metabolic diseases, are far from being fully understood. Epigenetics and metabolism trade interacting factors dynamically and reciprocally, while constantly being modulated by multifactorial external stimuli, unfolding correlations among different pathological conditions. In addition to immediate physiological effects like hormonal dysregulation and generation of adipose tissue, life style factors and food intake in particular may also affect the epigenome. For instance, there is strong evidence that nutritional habits can influence gene transcription epigenetically (van Dijk et al., 2015; Etchegaray and Mostoslavsky, 2016) and there are several studies claiming inheritance to the offspring (**Figure 1**) (Seki et al., 2012; Nicholas et al., 2013; Rando and Simmons, 2015). A recent genome wide analysis of DNA methylation in children born to parents affected by the Dutch famine showed that 70 years later several DNA regions associated with growth and metabolism were significantly differentially methylated, including genes involved in control of birth weight and LDL cholesterol in later life (Tobi et al., 2015).

Apart from the influence of dietary habits on epigenetic regulation of metabolism there are more societal factors such as sleep patterns, meal timing and work shifts that cause circadian misalignment. Even one night of sleep deprivation results in hypermethylation of various tissue specific clock genes, leading to increased insulin resistance and impaired glucose tolerance (Donga et al., 2010; Cedernaes et al., 2015; Fontana and Partridge, 2015; Morris et al., 2016). Furthermore, metabolites are the substrates used to form chromatin modifications and hold fundamental role in the activity of all biochemical pathways. It has been shown that metabolites deriving from various food sources can serve as substrates for transcription factors and histone modifying enzymes that then affect chromatin compaction, leading to more sequestered or exposed areas of the genome and to down- or upregulation of gene transcription, respectively (Lu and Thompson, 2012; Gut and Verdin, 2013; Fan et al., 2015).

On the other hand, there are several lines of evidence suggesting that epigenetics in turn could affect metabolism and disease (Crunkhorn, 2011; Heerboth et al., 2014; Kwak and Park, 2016). Hence, current efforts to examine epigenetic targets as future druggable candidates for metabolic diseases should be further increased. In this review, we will therefore focus on the complex and bidirectional interplay between epigenetics and metabolism and how this connection can be dysregulated in human disease.

## Rare Diseases: How Mutations in Genes of Epigenetic Regulators Are Associated With Metabolic Phenotypes and Developmental Transgenerational Diseases

Rare diseases are a group of human diseases defined by a very small amount of people affected by them. The vast majority of rare diseases still lack any therapy partially due to our very limited understanding of their pathomechanisms but also due to the lack of identified druggable targets. They encompass monogenetic as well as complex disorders, although not all rare diseases have a genetic background. Symptoms can vastly vary from birth dysplasia over to gradual atrophy and in multiple cases development of metabolic phenotypes.

Rett syndrome (RTT; OMIM 312750) is a rare progressive neurodegenerative disease affecting girls, characterized by reduced brain volume, speech and motor disabilities, breathing irregularities muscle deterioration throughout patient’s life and many metabolic complications. It is mainly caused by any of various mutations in one gene, coding for methyl-CpG-binding protein 2 (MECP2) (Kyle et al., 2016). MECP2’s central role is to regulate epigenetic imprinting and chromatin compaction. By selectively binding to methylated DNA, MECP2 regulates gene transcription through association with chromatin-remodeling complexes, including type I histone deacetylases (HDACs) (Justice et al., 2013). However, MECP2 influences many different biological pathways on multiple levels and the molecular pathways from gene to phenotype are currently not fully understood (Justice et al., 2013). Several studies though have begun to shed light on the mechanism behind RTT’s metabolic symptoms. In one study lipid metabolism was widely affected in MECP2 null mice, both in the brain and systemically (Buchovecky et al., 2013). Furthermore, abnormal mitochondrial function and morphology has been described in RTT patients and MECP2 mouse models (Kriaucionis et al., 2006). In recent studies MECP2 was found to regulate lipid homeostasis by recruiting the repressor complex, NCoR1/SMRT - HDAC3, to its lipogenesis targets in hepatocytes. Also, Mecp2 mutant mice develop fatty liver disease and dyslipidemia similar to HDAC3 liver-specific deletion (Kyle et al., 2016). These findings position MECP2 as an epigenetic component and regulator of metabolic homeostasis.

RTT is not the only rare disease with a direct link between epigenetics and metabolism. However, mutations in many of various epigenetic regulatory enzymes have historically first been associated with cancer. It has become clear that cell metabolism and cancer are tightly linked. For this reason, it is not surprising that many cancer-associated genes can also cause metabolic disease phenotypes without cancer symptoms. In particular, histone-lysine N-methyltransferase 2D (KMT2D), also known as MLL4 or MLL2 in humans and Mll4 or Mll2 in mice, is a major histone H3 lysine 4 (H3K4) mono-methyltransferase (Lee et al., 2013). It is primarily linked with acute myeloid leukemia, lung and colon cancer (Rao and Dou, 2015). However, mutations in KMT2D are also associated with Kabuki syndrome, which is a multiple congenital anomaly syndrome characterized by facial and skeletal anomalies, mild to moderate intellectual disability and postnatal growth deficiency (Van Laarhoven et al., 2015). In about 45–80% of patients’ mutations in the KMT2D gene are detected. While muscoskeletal abnormalities are the primary presentations of Kabuki syndrome, patients also suffer from metabolic deregulation symptoms later in life. Apart from the Kabuki syndrome, KMT2D has been shown to play a role in the development of congenital heart disease (Zaidi et al., 2013). Although the precise molecular mechanism underlying KMT2D’s impact on metabolism remains to be uncovered and might differ from disease to disease, it has been shown before that mutations in Mll2 result in insulin resistance and impaired glucose tolerance in mice (Goldsworthy et al., 2013). Furthermore, KMT2D mutant mice revealed features of mild non-alcoholic fatty liver disease (NAFLD), elevated cholesterol and triglycerides plasma levels. Another study identified KMT2D and KMT2C as regulators of the hepatic circadian clock and co-activators of the circadian transcription factors retinoid-related orphan receptor (ROR)-α and -γ, which have been implicated before in controlling lipid metabolism (Lau et al., 2008; Kim et al., 2015). In mice, KMT2D also acts as a coactivator of PPARγ within the liver to direct over-nutrition induced steatosis. Heterozygous Kmt2d+/- mice exhibit resistance to over-nutrition induced hepatic steatosis (Kim et al., 2016).

DNA methylation in mammals has long been implicated in the epigenetic mechanism of parental imprinting, in which selective expression of one allele of specific genes is governed by parental origin. To date, all imprinting control regions (ICRs) identified are differentially DNA methylated regions (DMRs) on the two parental chromosomes and are heritably maintained in the developing embryo. However, the role of histone modifications is less clear. DMRs are characterized by the asymmetrical accumulation of different histone modifications on the two parental chromosomes and a requirement for histone demethylation in order to establish germline DNA methylation has been identified at some ICRs (Ciccone et al., 2009). Imprinting disorders are a group of 12 diseases with overlapping clinical characteristics and common epigenetic patterns. They have been shown to affect growth, development and metabolism (Livingstone and Borai, 2014). In patients with Silver–Russell syndrome (SRS) the most common underlying disease-associated epigenetic change is loss of methylation on chromosome 11p15 (Schönherr et al., 2006). However, the molecular etiology remains unknown in a substantial cohort of patients. CpG islands represent the most interesting genomic regions for examining differences in methylation in SRS because methylation changes in these regions directly influence the local epigenetic environment to regulate gene expression. Interestingly, a recent study showed that CpG islands which are differentially methylated in SRS were enriched for genes linked to metabolism (Prickett et al., 2015). This could be a potential link between the observed metabolic symptoms of SRS patients, which include obesity, cardiovascular disorders and type II diabetes (Wakeling et al., 2017).

A number of imprinted genes contribute to the control of energy homeostasis and glucose regulated metabolism, including IGF2 and GRB10, DLK1 (Livingstone and Borai, 2014). Diseases associated with these genes serve to underline their importance for normal metabolic regulation, as exemplified by Beckwith–Wiedemann syndrome (BWS). BWS is an inherited condition characterized by excessive IGF2 expression, usually resulting from loss of imprinting. Hypoglycemia occurs in 50% of cases of BWS. This is caused by hyperinsulinism, rather than being a direct effect of Igf2, but the underlying β-cell defect is unknown (Sparago et al., 2004; Adachi et al., 2013). Epigenetic studies have shown that parental obesity can affect methylation of IGF2 of the fetus correlating these changes with birth weight and metabolic syndrome later in life (Hoyo et al., 2012; Soubry et al., 2013). In animal models, parental caloric restriction influences epigenetic regulation of Igf2 (Zhang et al., 2011). Transmission of a null Grb10 allele results in reduced adiposity, increased lean mass and improved glucose tolerance potentially via the Igf1 signaling and controlled insulin production (Mokbel et al., 2014). Interestingly, hypomethylation of the GRB10 locus has been implicated in the development of both SRS and Beckwith–Wiedemann syndromes (Scott and Moore, 2012; Koren and Palladino, 2016). Another potential factor governing a transgenerational epigenetic disease risk is DLK1 (also known as preadiposite factor 1) (Andersen et al., 2009). It is expressed in high level in preadipocytes, and downregulation of Dlk1 correlates with adipocyte differentiation *in vitro*. In an animal model a regulatory mutation causing partial loss of imprinting of the Dlk1-Dio3 cluster caused embryonic hypothyroidism in the offspring (Charalambous and Hernandez, 2013). The animals exhibit postnatal hypothyroidism and impaired brown tissue development due to overexpression of Dlk1, leading to lifelong hypothyroidism, obesity and glucose intolerance.

Taken together, it is becoming clear that DNA methylation, imprinting and chromatin modifications play crucial roles in the regulation of metabolism, and that their dysregulation in disease can cause devastating outcomes. Similarly, these epigenetic drivers of metabolism can be influenced by various non-genetic and extracellular cues, thereby mediating transgenerational effects on metabolism.

## Cancer: Epigenetic and Metabolic Interactions

Dysregulated metabolism is a hallmark of cancer (DeBerardinis and Chandel, 2016; Vander Heiden and DeBerardinis, 2017). Warbung was the first to describe an increased consumption of glucose by tumor tissues in comparison to normal cells (Warburg et al., 1927). High glutamine demand is another tumor characteristic, since glutamine is a key nitrogen input for the biosynthesis of cell components required for the fast growth typical of cancerous tissues (Eagle, 1955; Nicklin et al., 2009; DeBerardinis and Cheng, 2010). Due to their central role in cells, metabolic pathways are strictly regulated – and not just by the direct energy requirements of the cell. It has been demonstrated that the influx of glucose into cells is not only driven by the immediate bioenergetic needs of a cell but is also modified by extracellular stimuli (Grassian et al., 2011). Interestingly, tumor tissue might rely on nutrient adaptation and cell interactions in its microenvironment to obtain fast cell growth and proliferation. In line with this, it has been shown that in cancer, several metabolic pathways are dysregulated and are reprogrammed to favor tumor development even in the absence of essential nutrients (Boroughs and DeBerardinis, 2015).

Furthermore, the origin and the etiology of the tumor, the local nutrient availability and potency of metastasis, could excite activation of different metabolic pathways (Boroughs and DeBerardinis, 2015). Therefore, in the past years tumors started being profiled depending on their metabolic regulation (Yuneva et al., 2012; Wang et al., 2016). One caveat of metabolic tumor classification is that different areas of the same tumor show high metabolic heterogeneity, as shown by various screens setting out to identify metabolic changes for tumor categorization (Denkert et al., 2006; Li and Deng, 2017; Russell et al., 2017).

Despite the genetic and histological heterogeneity, tumor development seems to involve the common induction of a defined group of pathways to support core functions like anabolism, catabolism, and redox balance (Cantor and Sabatini, 2012). The rewired metabolism of cancer cells could be a cause or a consequence of the multiple changes in the epigenome which is tightly linked with nutrient and metabolite availability. Indeed, several studies have demonstrated that metabolic changes affect the epigenome to promote more cancerogenic alterations (Cai et al., 2011; Fan et al., 2015; Pietrocola et al., 2015; Feinberg et al., 2016; Roe et al., 2017). Furthermore, the epigenome may act in favor of uncoupling normal metabolic functions in favor of tumorigenic development.

A long list of metabolites play key roles as signaling molecules in the regulation of epigenetics in normal cells, including *S*-adenosyl methionine (SAM), which is required by DNA methyltransferases (DNMTs) and histone methyltransferases (HMTs) (Rea et al., 2000), flavin adenine dinucleotide (FAD) and 2-oxoglutarate (2-OG), which regulate lysine demethylases (KDM), acetyl-CoA, which is required for the addition of acetyl groups to histones by histone acetyl transferases (HATs) and nicotinamide adenine dinucleotide (NAD) which serves as a cofactor for class III histone deacetylases (Galdieri and Vancura, 2012). Metabolites therefore are involved in regulating all essential steps in establishing, modulating and removing epigenetic marks on histones.

Dysregulation of the cellular levels of these metabolites therefore establish direct routes of affecting the epigenome and subsequently impact cancer development (Kaelin et al., 2013; Mehrmohamadi et al., 2016). Mutations in the metabolic enzyme isocitrate dehydrogenase 1(IDH1) and IDH2 have been linked with glioblastoma and acute myeloid leukemia (Losman and Kaelin, 2013). These mutations cause altered enzymatic activity resulting in the production of the R-2-hydroxyglutarate (R-2HG) metabolite. High levels of R-2HG interfere with the function of dioxygenases requiring 2-oxoglutarate (2-OG) as a co-substrate. Dioxygenases play crucial roles in cells and include prolyl hydroxylases, cytosine hydroxylases, and histone demethylases. Inhibition of the TET DNA- and JmjC histone-demethylase families by increased levels of R-2HG influence gene expression in part via an altered epigenetic state, characterized by a failure to express differentiation programs (Dang et al., 2009; Figueroa et al., 2010). In this case even though the production of R-2HG is linked with metabolic deregulation, its effect on cancer fate seems to be of a non-metabolic nature, by causing a lack of differentiation.

Mutations in genes encoding fumarate hydratase (FH) and succinate dehydrogenase (SDH) have also been identified in many cancers. These mutations result in excess amounts of their products, which are both TCA cycle intermediates (Xiao et al., 2012). Even though they reprogram TCA cycle metabolism, similar to the IDH1/IDH2 mutations, the involvement of fumarate and succinate in cancer progression might be linked with extensive alterations in the epigenome as they also affect dioxygenase activity (Kaelin et al., 2013; Laukka et al., 2016).

Though mutations in genes related to cancer growth signaling pathways are at the moment outnumbering identified alterations in metabolic enzymes, increasing evidence demonstrates the importance of metabolic pathways in driving and supporting tumorigenesis. In particular, transcriptional factors, which are key players in androgen response, hypoxia, glycolysis, and lipid metabolism pathways, act synergistically with KDM3A to promote cancer (Wilson et al., 2016, 2017). Furthermore, in a study of 58 cancer cell types, DNA enhancer methylation was a strong predictor of cancer related gene expression and two thirds of the affected (hypomethylated/upregulated) genes had function in metabolic processes (Van Damme et al., 2016).

## Interventions and Future Aspects

As of June 2018, there were nine FDA-approved drugs that act primarily by targeting epigenetic mechanisms. Although most of these early drugs were approved for cancer treatment, an expanding portfolio of molecules targeting epigenetic factors in other diseases now is in preclinical and clinical development (Qi et al., 2016; Meighan-Mantha, 2017). Valproic acid is an HDAC inhibitor for psychiatric disorders which recently is being examined in clinical trials for different types of cancer. Although clinical trials for DNMTis and HDACis were first initiated 30 years ago, only in the past 5 years 2nd generation epigenetic drugs have entered in clinical trials stage. These novel drugs show higher potency and specificity targeting other enzymes, including bromodomain and extra terminal protein (BET), lysine-specific demethylase 1A (LSD1/KDM1A), mutant isocitrate dehydrogenase (IDH), histone methyltransferase (HMT) and protein arginine methyltransferase (PRMT). A recent but promising trend in the cancer treatment landscape is combination therapies of epigenetic drugs with other compounds (Dueñas-Gonzalez et al., 2014; Ahuja et al., 2016; Raynal et al., 2017). Epigenetic inhibitors could be the lead to treat multiple disease types apart from cancer. For example, ESR1 and ESR2, which are expressed in smooth vascular muscle cells, are usually hypomethylated in human atherosclerosis and folic acid deprivation is shown to play a role in endothelial dysfunction linked with cardiovascular disease and aging (Kim et al., 2007; Kok et al., 2015). As far as various neurological disorders are concerned, epigenetic modifications have long been implicated in the nervous system’s aging, plasticity and memory (Jakovcevski and Akbarian, 2012). ORY-2001 is the first Alzheimer’s related epigenetic drug that entered clinical phase I last year (Maes et al., 2016; Meighan-Mantha, 2017). Huntington’s (HD) is another neurodegenerative disease caused by a polyglutamine repeat sequence in the huntingtin protein, which inhibits HATs leading to a decrease of H3 and H4 histone acetylation. There are few recent studies showing that HDACi treatment halts effects of HD *in vivo* as well as other molecules that are still under investigation (Gray, 2010; Bürli et al., 2013; Duan, 2013). Other HDACi have also recently been used for the treatment of diabetes (Mau and Yung, 2014). However, it still remains elusive how epigenetic inhibitors affect the underlying disease-causing mechanisms. Novel ideas have arisen such as investigating metabolic checkpoint inhibitors for cancer treatment and reversal of progression (Scharping and Delgoffe, 2016). There are reasons to be optimistic that in the next years we will be able to better control the bidirectional relationship between epigenetic switches and metabolism and thereby address disease progression. Epigenetic interventions could improve the health state of people in reproductive age and halt disease development or transgenerational inheritance of the metabolic syndrome, which is critical to relief societal health burden. New epigenetics-based diagnostic tests may also help classify individuals with chronic diseases, prescribe pharmaceutical treatments fitting to the patient’s profile, minimize possible cytotoxicity or adjust dietary needs for health improvement of the individual. Furthermore, metabolic changes can be detected as early signs of various diseases including neurodegeneration (Kennedy et al., 2016).

## Conclusion

A growing body of evidence supports the intimate crosstalk between metabolism and epigenetics. Pathogenic conditions in which the interplay between metabolism and epigenetics is dysregulated, range from the rarest to the most common noncommunicable diseases of humanity. This bidirectional relationship could serve as the substratum of more complex organismal interrelations and intrarelations. It is therefore crucial that more fine-tuned research needs to be performed toward unraveling novel epigenetic players that regulate metabolism, and vice versa. Pinpointing the molecular mechanisms involved in key epigenetic enzyme regulation will support novel therapeutic approaches for various metabolic disorders. Furthermore, findings regarding epigenetic regulation of metabolism will reveal the untapped potential to modify pathological disease states.

## Author Contributions

ET wrote the article. AI and TD critically reviewed the article.

## Conflict of Interest Statement

The authors declare that the research was conducted in the absence of any commercial or financial relationships that could be construed as a potential conflict of interest.
