# Corrigendum: Epigenetics and Metabolism in Health and Disease

**DOI:** 10.3389/fgene.2020.00428

**Published:** 2020-04-23

**Authors:** Evangelia Tzika, Tobias Dreker, Axel Imhof

**Affiliations:** ^1^4SC AG, Translational Pharmacology, Munich, Germany; ^2^Faculty of Medicine, Ludwig Maximilians University of Munich, Munich, Germany; ^3^Protein Analysis Unit (ZfP), Biomedical Center, Ludwig Maximilians University of Munich, Munich, Germany

**Keywords:** epigenetics, metabolism, cancer, diabetes, HDACs, HATs, methyltransferases, demethylases

In the original article, there was an error in the Acknowledgments section. The selected icons used for Figure 1 with permission under the free license were sourced from two icon supply websites which have transferred ownership. As the current content on the websites have no relevance to our research, we have removed the respective links and the Acknowledgments section.

The authors state that this does not change the scientific conclusions of the article in any way. The original article has been updated.

